# Burnout in hospital healthcare workers after the second COVID-19 wave: Job tenure as a potential protective factor

**DOI:** 10.3389/fpsyg.2022.942727

**Published:** 2022-08-08

**Authors:** Helena Sofia Antao, Ema Sacadura-Leite, Ana Isabel Correia, Maria Luisa Figueira

**Affiliations:** ^1^Hospital Prof. Doutor Fernando Fonseca, EPE, Amadora, Portugal,; ^2^Faculdade de Medicina da Universidade de Lisboa, Lisbon, Portugal; ^3^Departamento de Saúde Ocupacional, Centro Hospitalar e Universitário de Lisboa Norte, Lisbon, Portugal; ^4^CISP - Centro de Investigação em Saúde Pública, Escola Nacional de Saúde Pública, Lisbon, Portugal

**Keywords:** burnout, depersonalization, emotional exhaustion, healthcare workers, job tenure, occupational health, personal accomplishment, protective factors

## Abstract

Burnout is an impactful occupational health phenomenon to which doctors and nurses have been more exposed during the COVID-19 pandemic. The objectives of this study were to measure the dimensions of burnout—emotional exhaustion, depersonalization and personal accomplishment—in a hospital healthcare population after the second COVID-19 wave and to study their association with sociodemographic variables and previous COVID-19 infection. We invited 112 healthcare professionals (doctors and nurses) who attended the occupational health department of a tertiary hospital in March–July 2021. Emotional exhaustion, depersonalization and personal accomplishment were measured by the Maslach Burnout Inventory. Linear-regression analyses were conducted to explore relationships between burnout dimensions and sociodemographic variables. Differences between groups according to previous COVID-19 infection were verified using the t-test and when appropriate the Mann–Whitney test (for continuous variables), the chi-square test and when appropriate the Fisher exact test (for categorical variables). We surveyed 106 subjects (95% response rate). High emotional exhaustion and depersonalization were reported by 33.0 and 18.4% of participants, respectively; 21.4% reported low personal accomplishment. Job tenure was associated with depersonalization and personal accomplishment. For each 1-year increase in job tenure, depersonalization decreases 0.14 (95% CI [−0.23, −0.04]) and personal accomplishment increases 0.16 (95% CI [0.06, 0.25]). Gender was associated with emotional exhaustion (being male increases emotional exhaustion 5.62-fold [95% CI: 1.33; 9.92]). The prevalence of high emotional exhaustion, high depersonalization and low personal accomplishment after the second COVID-19 wave was relevant and should not be overlooked. Our findings suggest that job tenure may play a protective role in healthcare workers’ burnout.

## Introduction

Healthcare workers’ mental health was profoundly affected by the COVID-19 pandemic (Chirico et al., 2021; De Sousa et al., 2021; Teo et al., 2021; Aymerich et al., 2022). Burnout, specifically, is an impactful occupational health phenomenon to which doctors and nurses have been more exposed during this period and may have long-term psychological implications (Barello et al., 2020; Duarte et al., 2020; Conti et al., 2021; Galanis et al., 2021; Leo et al., 2021).

Burnout is a prolonged response to chronic emotional and job-related stress (Maslach et al., 2001) characterized by a combination of emotional exhaustion (EE), depersonalization (DP) and low personal accomplishment (PA) that affects workers worldwide (Maslach, 2017). The 11th Revision of the International Classification of Diseases (ICD-11) acknowledges it as an important occupational phenomenon and describes burnout as “a syndrome conceptualized as resulting from chronic workplace stress that has not been successfully managed” (World Health Organization, 2019).

Although it can occur in any kind of profession, it was originally described and discussed as a human service sector problem especially in healthcare, education, social work, psychotherapy, legal services, and law enforcement, where bureaucratization and lack of reciprocity often replace the workers’ initial idealism by feelings of frustration and disillusion (Burnout, 2017).

Organizational and team-level constraints create stress-inducing situations for healthcare workers that compromise their well-being and put them at higher risk for burnout (Ramos et al., 2020). Burnout in healthcare workers may result not only in harmful consequences for their health but also in decreased quality of patient care. Brain research has shown that uncontrollable stress reduces prefrontal cortex activity—a brain area responsible for self-regulation—which could explain the reduced motivation, unprofessional behavior, and ineffective communication with patients frequently found in burned out physicians (Arnsten and Shanafelt, 2021). These behaviors make healthcare professionals feel they are not fulfilling their role adequately and, as workplace stress evolves, this loop of feelings produces dysfunctionality (Misiolek-Marín et al., 2020). For these reasons, particular attention has been dedicated to understanding burnout risk and protective factors in this professional setting.

The studies carried out before the pandemic had already found an increased risk of burnout in female gender, younger age, lonely partnership status (being single, divorced, separated or widowed), having children and low job satisfaction(Lin et al., 2020; Zborowska et al., 2021). Additionally to these, burnout research that took place during the pandemic, identified other risk factors as decreased social support, low family and colleagues’ readiness to cope with COVID-19 outbreak, working in the frontline, being a resident, experiencing the loss of patients, increased workload, shifts lasting 8 h or more, onsite redeployment and low level of specialized training (El-Hage et al., 2019; Tan et al., 2020; Conti et al., 2021; Galanis et al., 2021; Jalili et al., 2021; Lluch et al., 2022).

Hardiness, resilience, self-efficacy, perceived social support, job satisfaction and satisfaction with life were found to be protective factors for burnout during the COVID-19 pandemic (Duarte et al., 2020; Wu et al., 2020; Galanis et al., 2021; Vagni et al., 2021; Zotović et al., 2022) adding to adequate sleeping hours and days off, safety conditions, working environment, mutual support networks/coping strategies, patients’ support and gratitude, payment and policies governing professional practice (Wisetborisut et al., 2014; Converso et al., 2015; Brigham et al., 2018; Giménez et al., 2021) that had been described beforehand.

Although shorter job tenures have been associated with higher levels of burnout in studies carried out during the COVID-19 period (Hwang et al., 2021; Zhou et al., 2021), its impact has not been as extensively studied as other risk and protective factors. Despite co-varying and being both time related, age and job tenure have been described as distinct variables, and some authors refer to job tenure as a more consistent and stable predictor of job satisfaction than age (Bedeian et al., 1992), which reinforces the need of studying its effects on burnout in more depth. Although the number of years that workers have already spent at their jobs cannot be changed, job tenure is a modifiable variable when looked at prospectively, since both employees and employers can choose to extend it by not terminating the job relationship they share.

COVID-19 infection and mortality among healthcare workers followed that of the general population around the world, with 152,888 infections reported during the early phases of the pandemic (Bandyopadhyay et al., 2020). COVID-19 infected individuals may experience long-lasting symptoms for several months after the acute phase of the disease that are not limited to the respiratory system and include, among others, fatigue, headaches, depression and anxiety (Michelen et al., 2020). Post-mortem studies have found a high expression of the angiotensin-converting enzyme 2 (ACE2) transmembrane receptor in the amygdala (Lukiw et al., 2022) and the presence of SARS-CoV-2 RNA in one or more brain regions including the amygdala (Serrano et al., 2021). Burned-out subjects have a lower ability to down-regulate negative emotions that has been explained on the grounds of a weaker functional connectivity observed in MRI studies between the amygdala and other brain structures (Golkar et al., 2014). These facts raise the hypothesis of a higher susceptibility of COVID-19 infected subjects to the negative effects of burnout, but its supporting evidence is limited. One study described a positive relationship between hospitalization for COVID-19 symptoms and posttraumatic stress (Luceño-Moreno et al., 2020) and another one detected more burnout in nurses who had a positive COVID-19 test and did not want to work voluntarily during the outbreak (Murat et al., 2021). There is therefore a knowledge gap in the effects of COVID-19 infection on burnout levels and emotional balance in the specific population of healthcare workers that needs to be addressed.

The objectives of our research were to measure the levels of the three burnout dimensions among healthcare workers in a hospital setting during the COVID-19 pandemic and to study their association with sociodemographic variables and previous COVID-19 infection.

## Materials and methods

### Study design and source population

This was an observational, cross-sectional study, with data collected in pre-defined periods between March and June 2021 at the occupational health department of a Portuguese tertiary teaching hospital. Doctors and nurses who attended work medicine appointments according to the established occupational health surveillance plan of the hospital were the source population of the study.

We used a convenience sampling method for recruitment, personally inviting the source population individuals to participate.

### Instruments of measurement and variables

The questionnaire included items about demographics (age, gender, professional category, partnership, and parental status) and other characteristics (medical specialty, team coordination, nocturnal work, exercising habits, practice of relaxation techniques, and previous COVID-19 infection).

Participants’ burnout levels were determined using the Portuguese validated version of the 22 items Maslach Burnout Inventory—Human Services Survey (MBI-HSS) (Maslach, 1996; Cardoso et al., 2002). MBI-HSS is the most widely used instrument to measure burnout among healthcare professionals that addresses three burnout dimensions: EE (which measures feelings of being emotionally overextended and exhausted by one’s work), DP (which measures an unfeeling and impersonal response toward recipients of services, care or instruction) and PA (which measures feelings of competence and successful achievement in one’s work).

In the definition of low, average, or high levels of each dimension of the MBI-HSS, we have used the following cut-offs: EE: low, ≤13; average, 14–26; high, ≥27; DP: low, ≤5; average, 6–9; high, ≥10; PA: low, ≤33; average, 32–39; high, ≥40 (Soler et al., 2008).

We used the individual dimension scores as continuous data to evaluate their relationship with sociodemographic variables (Dyrbye et al., 2009).

Job tenure was defined as the number of years the respondent had been working at the hospital where the study took place. Individuals were classified as team coordinators if they declared to coordinate a team on a permanent or occasional basis. Respondents who accomplish at least one night shift per month were considered to do nocturnal work. Participants were classified as partnered if declared to be married or similar (living with someone with whom they had an intimate relationship) and having a parental status ≤ 12 if they lived with one or more children aged 12 or less. Exercise was defined as practicing the following activities at least twice a month: walking, running, cycling, kick boxing, cardio training, muscle strengthening, stretching, tennis, padel, swimming and gardening. If practiced at least once a month, the following relaxing activities were considered: meditation, yoga, relaxing massage and playing a musical instrument.

### Statistical analysis

Descriptive analyses were conducted to examine the participants’ characteristics and outcome variables. Means, medians, interquartile ranges and standard deviations were calculated for continuous variables. Frequencies and percentages were obtained for categorical variables.

Three outcomes were considered, corresponding to the three dimensions of burnout. Linear regression was used to assess the association between burnout dimensions and gender, professional category, job tenure, team coordination, nocturnal work, partnership status, parental status ≤ 12, practice of exercise, practice of relaxation techniques and COVID-19 infection status.

We excluded age from our analysis since this variable was strongly correlated with job tenure.

We have adjusted the association between team coordination and DP for job tenure as we considered it a confounding factor in the relationship between those two variables (healthcare workers with longer job tenures are more often chosen to coordinate teams).

Differences between the group who had COVID-19 infection in the previous 12 months and the non-infected group were assessed for statistical significance using the t-test and when appropriate the Mann–Whitney (for continuous variables), the chi-square test and when appropriate the Fisher exact test (for categorical variables). All reported *p* values are two-tailed and *p* < 0.05 was considered statistically significant.

All analyses were performed using IBM SPSS Statistics v.26.

### Ethics statement

The study was conducted according to the guidelines of the Declaration of Helsinki and approved by the Ethics Committee of CHULN (Centro Hospitalar e Universitário de Lisboa Norte) and CAML (Centro Académico de Medicina de Lisboa). Approval was also obtained from the CHULN administration board. The study objectives and goals were explained to the healthcare workers invited to participate. The informed consent form was provided to those who showed interest in participating. Participants’ written informed consent was obtained prior to the study procedures.

## Results

Of the 112 healthcare workers invited, 106 (51 nurses and 56 doctors) accepted to participate (95% response rate).

Medical specialties and departments to which doctors and nurses belonged included anesthesiology, cardiology, dermatology, ear nose and throat, endocrinology, gastroenterology, gynecology, hematology, imageology, immunoallergology, intensive care, internal medicine, laboratory medicine, neurology, occupational medicine, ophthalmology, pediatrics, pharmacology, psychiatry, pulmonology, rheumatology, surgery, and urology.

High EE and DP scores were reported by 33.0% and 18.4%, respectively; 21.4% reported a low PA score.

Table 1 summarizes the main participants’ sociodemographic characteristics and burnout rates.

**Table 1:** Sociodemographic characteristics and burnout rates (*N*= 106).

Age, median, IQR	35.0 (28.0, 48.3)
**Gender, n° (%)**	
Male	32 (30.2)
Female	74 (69.8)
**Professional category, n° (%)**	
Nurses	51 (48.1)
Doctors	55 (51.9)
Job tenure in years, median, IQR	9.5 (0.9, 22.8)
Less than 1 year, *n*° (%)	26 (24.5)
1–5 years, *n* (%)	21 (19.8)
6–10 years, *n* (%)	7 (6.6)
11–20 years, *n* (%)	22 (20.8)
More than 20 years, *n* (%)	30 (28.3)
Team coordinators, *n* (%)	21 (19.8)
Nocturnal work, *n* (%)	52 (49.1)
Partnered, *n*° (%)	69 (65.1)
Parental status ≤ 12, *n* (%)	32 (30.2)
Exercise, *n* (%)	53 (50.5)
Relaxation, *n* (%)	13 (12.3)
Covid-19 infected, past 12 M, *n* (%)	28 (26.4)
**Burnout dimensions**	
**Emotional exhaustion (N = 99)**	
Mean	19.7
Median	19.5
IQR	12.0, 26.0
% High score	33.0
**Depersonalization (N = 104)**	
Mean	5.3
Median	3.0
IQR	1.0, 8.0
% High score	18.4
**Personal accomplishment (N = 103)**	
Mean	37.4
Median	38.0
IQR	33.0, 41.0
% Low score	21.4

Gender was associated with EE; being male increases EE 5.62-fold (95% CI [1.33; 9.92]).

Job tenure was associated with the DP and PA dimensions of burnout. For each 1-year increase in job tenure, DP decreases 0.14 (95% CI [−0.23, −0.04]) and PA increases 0.16 (95% CI [0.06, 0.25]).

Table 2 depicts the relationship between the three burnout dimensions and the independent variables.

**Table 2:** Factors related to burnout dimensions on regression analysis (N = 106).

	Emotional exhaustion (*N* = 99)			Depersonalization (*N* = 104)			Personal accomplishment (*N* = 103)		
	UnStd B	C.I.	Value of *p*	UnStd B	C.I.	Value of *p*	UnStd B	C.I.	Value of *p*
Gender (male/female)	5.62	[1.33, 9.92]	0.011^*^	−0.87	[−3.48, 1.74]	0.509	−2.03	[−4.64, 0.59]	0.127
Prof category (physician/nurse)	1.36	[−2.78, 5.50]	0.516	−0.48	[−2.88, 1.92]	0.692	−0.38	[−2.83, 2.06]	0.756
Job tenure	0.06	[−0.11, 0.23]	0.499	−0.14	[−0.23, −0.04]	0.005^*^	0.16	[0.06, 0.25]	0.002^*^
Team coordination	0.26	[−5.47, 5.99]	0.929	1.71	[−1.53, 4.95]	0.297	−1.41	[−4.72, 1.90]	0.400
Nocturnal work	2.39	[−1,73, 6.51]	0.252	−2.03	[−4.40, 0.33]	0.090	0.21	[−2.24, 2.66]	0.865
Partnered	0.49	[−3.95, 4.93]	0.828	1.51	[−1.01, 4.02]	0.237	−1.92	[−4.48, 0.63]	0.139
Parental status ≤ 12	1.80	[−2.67, 6.26]	0.427	−0.10	[−2.73, 2.53]	0.941	−1.59	[−4.20, 1.03]	0.231
Exercise	−0.82	[−5.01, 3.36]	0.697	1.26	[−0.62, 3.12]	0.183	1.01	[−1.44, 3.46]	0.416
Relaxation	−3.23	[−9.36, 2.90]	0.299	−3.78	[−7.59, 0.33]	0.052	0.53	[−3.63, 3.74]	0.977
Covid infected, past 12 M	−2.40	[−6.99, 2.19]	0.302	−1.56	[−4.27, 1.15]	0.256	0.12	[−2.63, 2.87]	0.932

* Statistical significance, 95% CI.

**Figure 1:**
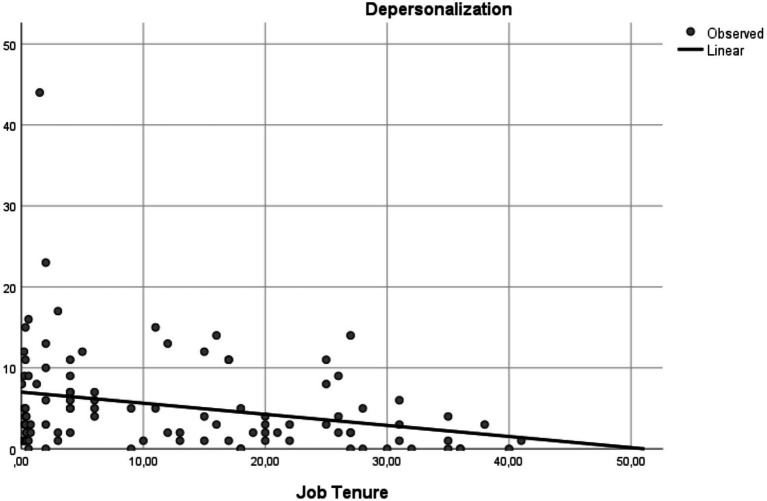
Effect of job tenure on depersonalization: regression analysis curve estimation.

**Figure 2:**
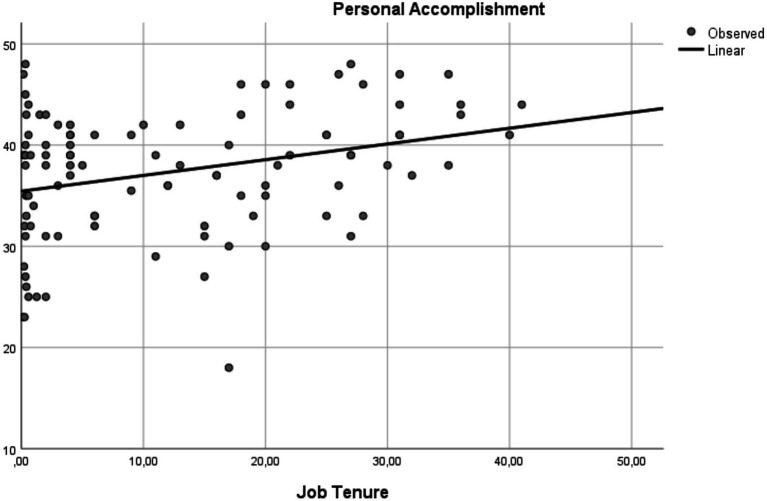
Effect of job tenure on personal accomplishment: regression analysis curve estimation.

Figures 1, 2 illustrate the associations found between job tenure and the burnout dimensions DP and PA.

Table 3 depicts the burnout dimensions’ scores and the percentages of high (EE and DP) or low (PA) scores of the study sample, according to previous COVID-19 infection status. No differences were found between the two groups.

**Table 3:** Burnout in COVID-19 Infected and Not COVID-19 Infected, past 12 M (N = 106).

Burnout dimensions	Infected (*N* = 28)	Not Infected (*N* = 78)	Value of *p*
**Emotional exhaustion (N = 99)**			
Mean	18.0	20.4	0.322
Median	17.5	21.0	
IQR	9.3, 25.8	12.0, 26.8	
% High score	28.6	34.7	0.640
**Depersonalization (N = 104)**			
Mean	4.1	5.7	0.453
Median	2.0	3.5	
IQR	1.0, 6.0	1.0, 8.0	
% High score	14.8	19.7	0.774
**Personal accomplishment (N = 103)**			
Mean	37.3	37.4	0.614
Median	38.0	39.0	
IQR	33.0, 41.5	33.0, 41.0	
% Low score	21.4	21.3	1.000

## Discussion

The prevalence of high burnout levels found in our study (33.0% for high EE, 18.4% for high DP and 21.4% for low PA) was relevant and should not be overlooked.

Direct comparisons to similar studies are difficult to make rigorously due to the differences in source populations and instruments used to measure burnout. Compared to Galanis et al., who have also used the MBI to measure burnout levels during the COVID-19 pandemic, the burnout prevalence found in our study is slightly lower for EE and higher for DP and PA, whereas compared to Ghahramani et al. (2021), whose systematic review included studies carried during the pandemic that used different instruments, our burnout prevalence is lower for the three dimensions.

On top of differences in source populations and MBI measurement instruments, the differences found across studies may be also due to heterogeneity in variables difficult to control like the efficacy of occupational health interventions, specialized training and psychological support provided to healthcare workers as well as the adjustments to healthcare staff schedules and tasks that were frequently made during the pandemic period. The possibility that physician burnout was higher than usual in some of its dimensions may have been due to the trial and error of treatment for an unknown virus and the uncertainty of the results.

In this study, we found associations between burnout levels and two sociodemographic variables: gender and job tenure.

The positive relationship between male gender and EE found in our study outlies from the available literature. Most studies carried in healthcare workers report higher levels of burnout in females (Abraham et al., 2020; Duarte et al., 2020; Gambaro et al., 2021). A metanalysis on male–female differences in burnout, including but not limited to healthcare workers, reported higher DP in males and higher EE levels in females (Purvanova and Muros, 2010). During the initial phase of the COVID-19 pandemic, higher DP levels have been found in male healthcare workers but still, contrary to our findings, higher EE rates in females (Brera et al., 2021).

Some authors have pointed out that the common assumption that females are more vulnerable to burnout than males may result in men not getting adequate attention and care when experiencing burnout which can ultimately reverse the expected outcomes. Another possible explanation for our finding is that during schools’ lockdowns more mothers than fathers could have been absent from work for childcare and, therefore, more protected from work exhaustion.

The inconsistency found in the literature about whether burnout increases or decreases with the length of tenure (Green et al., 2014; Abraham et al., 2020) may arise from the variability of environmental factors across organizations whose confounding effect is difficult to control. It may also be due to employees’ mixed attitudes toward negative environments: not leaving their jobs despite feelings of burnout and low satisfaction (conservative, low risk attitude, fear of the uncertainty and turmoil of challenges faced by healthcare institutions during pandemic peaks) or leaving in search of a better working life.

Some authors who have previously found an impact of job tenure over burnout interpreted it based on differences in social competencies and ways of coping with stress.

Choosing to stay with the same employer or to move to a new job opportunity is a trade-off between improved security and getting a better job. Staying long years in the same job may be considered an unambitious attitude toward career (Carmichael, 2015) and is sometimes perceived, especially by younger employees, as a hinder to getting job offers from the best employers.

The significant association between job tenure and DP/PA burnout found in our study can have several explanations and implications.

The “healthy worker effect” concept applies to these findings. Workers who were unable to cope with high burnout circumstances were more likely to have left and, therefore, had a lower probability of being included in the study. Workers who score burnout questionnaires more favorably may be less prone to give up their jobs for stress related reasons. This interpretation is supported by previous research that found a beneficial effect of high resilience on worker’s perceptions and psychological responses to stress (Shatté et al., 2017).

It may also be related to a time dependent desensitization toward workplace adverse situations and to a decrease in the frequency of those adverse situations resulting from status and respect gained throughout the years. The effectiveness of adverse situations management progresses along a learning curve.

In what concerns the PA dimension, it is understandable that healthcare workers with longer tenures have more opportunities to witness the outcomes of their work that, in the case of healthcare workers, generally consists of meaningful and fulfilling activities around improving patients’ health both through individual and teamwork approaches.

For short tenure workers who experience high burnout levels these results deliver a “things will get better” message that may dissuade them to leave and contribute to increased resilience that will pay off in the medium to long run.

Turnover is considered a negative internal indicator by most organizations and the common belief is that employees leave their jobs due to dissatisfaction and other negative reasons. Several studies describe burnout as a reason or mediator of high turnover rates among healthcare workers that should be kept at low levels to increase employee retention (Neale, 1991; Leiter and Maslach, 2009; Mahoney et al., 2020; Shields et al., 2020; Darbyshire et al., 2021; Kim and Kim, 2021).

Our findings underline the importance and potential benefits of reducing burnout levels also from the employer’s point of view.

Previous research identified the practice environment as the most common predictor of physicians’ burnout. Commitment to increase doctors and nurses’ tenures can help hospitals to perform better in indicators related to staff occupational health and, consequently, to improve the quality of care provided to patients.

Employees and employers are the main decision makers for job continuation or termination which makes job tenure a prospectively modifiable variable. Taking advantage of its protective effects over burnout is therefore under the control of these two players.

Our study did not replicate the common findings of higher burnout levels in nurses than in doctors (Ramirez-Baena et al., 2019). These higher levels have previously been explained as a generalized high nurse-to-patient ratio, closer proximity to patients, high workload, long working days, and shift work (O’Mahony, 2011). The absence of differences in burnout levels between the two professional categories in our study may be due to similar job demands and challenges in the center where the study took place. Doctor-to-patient ratios are also high, doctors are similarly close to patients and workloads are high for both categories. These similarities may have been favored by the COVID-19 pandemic working environment where the need to ensure optimal patient care tended to blur the differences between the roles and working paces of doctors and nurses belonging to the same teams.

We did not find an effect of team coordination over burnout. The research about how burnout levels vary with career stage and hierarchical position is controversial. Excessive workload, conflicts’ mediation, and lack of support from superiors have been described as contributors to burnout in senior positions (Membrive-Jiménez et al., 2020) but these findings are not necessarily translated into higher burnout levels compared to lowest levels in hierarchy (Luan et al., 2017), which was the case of our study findings. This may be due to heterogeneity in specific responsibilities, management expectations and job demands.

Our results are not in line with the published evidence that finds burnout to be more frequent among workers whose shifts include nocturnal work. We interpret this discrepancy on the grounds of the working schedules fluctuations that took place during the COVID-19 pandemic like frequent quarantines and alternate absences of teams’ members that were put in place to avoid a reduction in healthcare resources that would result from massive outbreaks among healthcare professionals.

We did not find an effect of being partnered or having children over burnout levels. The protective role of not living alone depends on several factors including the quality of family relationships (Mikolajczak et al., 2018). It also depends on the household composition and even for the same variable, for instance “having children” (that we mention as a protective factor documented in studies carried out before the COVID-19 pandemic), contradictory results are found in studies carried out during the COVID-19 pandemic [(Maunder et al., 2021) have found that psychological distress increases over time in those with children whereas (Cabezon et al., 2021) state that living with one or more children stands out as a protective factor] that can be due to heterogeneity in children care available, among other factors.

The protective role of exercise over burnout has been found mainly in studies where participants follow a strict pattern [e.g., “aerobic and strength training practiced 3 times a week or in alternate days as per ACSM prescribed exercise guidelines” (Ferreira and Rosales-Ricardo, 2022) or “vigorous physical exercise where a rapid heartbeat is achieved like intense swimming, running, cycling” (Heuse et al., 2021)]. This intensity of practice is not common because integrating vigorous exercise into everyday life is time-consuming and demands a self-regulatory effort. The number of individuals exercising according to those patterns is low in the general population and we aimed to assess whether the mild to moderate exercise that can be smoothly integrated in busy working schedules could make some difference. No protection over burnout can be expected from mild to moderate exercise as we have defined it.

There is conflicting evidence about the efficacy of relaxation techniques in burnout management. A Cochrane review (Ruotsalainen et al., 2014) found a 23% reduction in stress levels achieved by both mental and physical relaxation compared to no intervention in 17 out of 58 studies (low-to moderate-quality evidence). In the remaining 41 studies, no relationship was found. Our study results do not bring additional support to the pro side of this discussion.

No conclusions could be drawn from our study in what concerns long-lasting effects of COVID-19 infection on healthcare workers’ burnout levels or whether COVID-19 infected healthcare workers have a higher susceptibility to the negative effects of burnout. This may be due to heterogeneity in time after recovery and disease severity in a small sample of 28 infected subjects. This topic should undergo further research carried out on larger samples.

### Limitations and future research

Although its relevant findings, our study has some limitations such as being single centered and cross-sectional, having a small convenience sample size and not fully capturing workload fluctuations during the pandemic. Generalizations to hospital populations of healthcare workers should therefore be cautious.

Having in mind that employees’ reactions are not only job specific but also time dependent, the potential protective role of job tenure on burnout should be further studied in longitudinal studies, that will additionally test whether this effect prevails after the COVID-19 pandemic. As individual resilience, good coping skills and job satisfaction may influence job tenure, we suggest these variables are controlled in future studies. This will help to clarify whether job tenure is a protective factor by itself or a mediator between these variables and burnout.

## Conclusion

The prevalence of high EE, high DP and low PA after the 2nd COVID-19 wave was relevant and should not be overlooked. Our findings suggest that job tenure may play a protective role in burnout for healthcare workers, specifically in its DP and PA dimensions.

Since job tenure is a modifiable variable in workers’ lives from a prospective point of view, our findings can be an incentive to turnover reduction both to employees and to employers. More studies are needed to better understand the mechanisms involved.

The effects of COVID-19 infection on healthcare workers’ mental health and burnout levels are still poorly known and should undergo further research.

## Data availability statement

The raw data supporting the conclusions of this article will be made available by the authors, without undue reservation.

## Ethics statement

The studies involving human participants were reviewed and approved by Comissão de Ética do CHULN e do CAML. The patients/participants provided their written informed consent to participate in this study.

## Author contributions

HA, ES-L, and MF: study conception and design. HA and AC: data collection. HA, ES-L, AC, and MF: analysis and interpretation of results. HA: draft manuscript preparation. All authors contributed to the article and approved the submitted version.

## Funding

The article processing fee has been supported by Lundbeck Portugal.

## Conflict of interest

The authors declare that the research was conducted in the absence of any commercial or financial relationships that could be construed as a potential conflict of interest.

## Publisher’s note

All claims expressed in this article are solely those of the authors and do not necessarily represent those of their affiliated organizations, or those of the publisher, the editors and the reviewers. Any product that may be evaluated in this article, or claim that may be made by its manufacturer, is not guaranteed or endorsed by the publisher.
